# Ecotoxicity of as‐synthesised copper nanoparticles on soil bacteria

**DOI:** 10.1049/nbt2.12039

**Published:** 2021-03-30

**Authors:** Purnima Sharma, Dinesh Goyal, Bhupendra Chudasama

**Affiliations:** ^1^ Department Biotechnology Thapar Institute of Engineering and Technology Patiala India; ^2^ School of Physics and Materials Science Thapar Institute of Engineering and Technology Patiala India; ^3^ Thapar‐VT Center of Excellence in Emerging Materials (CEEMS) Thapar Institute of Engineering and Technology Patiala India

## Abstract

Release of metallic nanoparticles in soil poses a serious threat to the ecosystem as they can affect the soil properties and impose toxicity on soil microbes that are involved in the biogeochemical cycling. In this work, in vitro ecotoxicity of as‐synthesised copper nanoparticles (CuNPs) on *Bacillus subtilis* (MTCC No. 441) and *Pseudomonas fluorescens* (MTCC No. 1749), which are commonly present in soil was investigated. Three sets of colloidal CuNPs with identical physical properties were synthesised by chemical reduction method with per batch yield of 0.2, 0.3 and 0.4 gm. Toxicity of CuNPs against these soil bacteria was investigated by MIC (minimum inhibitory concentration), MBC (minimum bactericidal concentration), cytoplasmic leakage and ROS (reactive oxygen species) assay. MIC of CuNPs were in the range of 35–60 µg/ml and 35–55 µg/ml for *B. subtilis* and *P. fluorescens* respectively, while their MBC ranged from 40–70 µg/ml and 40–60 µg/ml respectively. MIC and MBC tests reveal that Gram‐negative *P. fluorescens* was more sensitive to CuNPs as compared to Gram positive *B. subtilis* mainly due to the differences in their cell wall structure and composition. CuNPs with smaller hydrodynamic size (11.34 nm) were highly toxic as revealed by MIC, MBC tests, cytoplasmic leakage and ROS assays, which may be due to the higher active surface area of CuNPs and greater membrane penetration. Leakage of cytoplasmic components and generation of extra‐cellular oxidative stress by reactive oxygen species (ROS) causes cell death. The present study realizes in gauging the negative impact of inadvertent release of nanoparticles in the environment, however, in situ experiments to know its overall impact on soil health and soil microflora can help in finding solution to combat ecotoxicity of nanoparticles.

## 1 INTRODUCTION

Over the past few years there has been a growing interest in the synthesis of copper nanoparticles (CuNPs) because of their potential applications in biology as nanomedicine. CuNPs and their complexes are now used as antibacterial, antiviral and anti‐fouling agents [1]. They have also demonstrated anti fungal activities. CuNPs help in collagen cross‐linking and in bone matrix formation [1]. The United States Environmental Protection Agency has registered copper as antimicrobial agents against specific harmful bacteria (European Copper Institute, 2008) [2]. Copper is an essential trace element that plays a vital role in metabolic and physiological processes in animals and plants but can cause acute toxicity if consumed/exposed in excess beyond safe limits [2]. Furthermore, these limits are being defined for bulk copper. Unlike the other properties of matter, antimicrobial activities are also expected to enhance when the particle size of copper reduces from bulk to nano [3, 4]. Antibacterial action of nanoparticles involves release of free ions, which can greatly affect the other living organisms [5, 6, 7]. Leaching of ions from nanoparticles contaminates soil and groundwater where they migrate into surface and interacts with living systems. Exposure of microbial communities to nanoparticles can cause hindrance in nutrient recycling, which can alter the productivity of ecosystems [7, 8].

Environmental accumulation of nanoparticles is a matter of concern as they are threat to microbial communities [9, 10]. Toxicity of nanoparticles towards soil organisms and their effect on soil properties is critical as 28% of total nanoparticle production is expected to end up into the soil [7, 11]. Leaching of nanoparticles alters the soil properties like pH, cation exchange capacity, porosity, organic matter content, availability of plant nutrients and soil enzyme activities [7, 11]. Ng and Coo [12] reported that mixing 2% of copper oxide nanoparticles or aluminium oxide nanoparticles reduced the hydraulic conductivity of kaolinite clays, which causes pore blocking in soil, whereas mixing of 6% of copper oxide nanoparticles or aluminium oxide nanoparticles with kaolinite clay induced shrinkage and helped the soil to maintain its aggregated structure under dry condition [Coo et al. [13]]. Collions et al. [14] and Fernandes et al. [15] reported that both copper nanoparticles (CuNPs) and copper oxide nanoparticles (CuONPs) caused significant but different changes in the structure of microbial community in farm topsoil and rhizosphere soil. Eivazi et al. [16] reported that as compared to the long‐term exposure, the short‐term exposure of silver nanoparticles reduced the enzyme activity in soil. Josko et al. [17] reported that ZnO and CuO nanoparticles lead to increase in the number of bacteria, fungi along with soil dehydrogenase activity in different soils.

Makama et al. [18] reported size‐dependent toxicity of polyvinylpyrrolidone (PVP) coated silver nanoparticles (AgNP) on earthworm (*Lumbricus rubellus*) through impaired reproduction and decrease in cocoon production by accumulation of silver ions. In another study Topuz and Gestal reported higher toxicity of PVP coated AgNPs on Annelid worms (*Enchytraeus crypticus*) by decreasing the organic carbon content of Lufa soils [19]. Volkar et al. [20] reported the toxicity of silver nanoparticles on freshwater snail (*Sphaerium corneum*) through reduced reproduction rate, ROS formation and altered activity of antioxidant enzymes. Gautam et al. [21] studied the ecotoxicity of copper oxide nanoparticles on the most common earthworm (*Metaphire posthuma*) present in Indian subcontinent and reported reduced population density with disturbed immunity. Therefore, it is essential to evaluate the toxicological properties of nano‐sized copper nanoparticles, so that the exposure limits can be defined for their safe usage and application [7]. Amongst different soil bacteria, *B. subtilis* and *Pseudomonas* play critical role in degradation of pollutants and elemental recycling [22]. Some strains of *B. subtilis* can mineralise explosives into harmless compounds and species of *Pseudomonas* are involved in bioremediation of chemical pollutants [23]. Very little information is available in the literature on the effect of nanoparticles on bacteria that are commonly present in the soil. Khurana et al. [3] reported that low concentration of silver and copper nanoparticles could impart severe toxicity on soil bacteria (*B. subtilis* and *P. fluorescens)*. Similar observation was reported by Yerukala and Bokka who studied the antimicrobial potential of copper nanoparticles on two plant biocontrol agents (*P. fluorescens* and *B. subtilis*). They reported higher sensitivity of copper nanoparticles against *P. fluorescens* as compared to *B. subtilis* [24]. In another study, Gajjar et al. [25] reported the antimicrobial activities of commercial nanoparticles (Ag, CuO and ZnO) against the environmental soil microbe, *Pseudomonas putida* KT2440. None of these studies provides an insight into the underlying mechanism responsible for observed ecotoxicity.

Despite multiple reports on ecotoxicity of CuNPs on soil and soil microbes, no attempts have so far been made to understand the effect of per batch yield of CuNPs on their ecotoxicity. Per batch yield of CuNPs is an important parameter, which affects the physical and chemical properties of nanoparticles. In this study we have investigated the ecotoxicity of three sets of identical colloidal copper nanoparticles (CuNPs) with per batch yield 0.2, 0.3 and 0.4 gm on soil bacteria *Bacillus subtilis* (MTCC No. 441) and *Pseudomonas fluorescens* (MTCC No. 1749). Their toxicity was evaluated by measuring the minimum inhibitory concentration (MIC) and the minimum biocidal activities (MBC). An insight into the nanoparticle‐bacterial interaction and underlying mechanism responsible for the observed ecotoxicity was provided by measuring protein and sugar leakages and ROS (reactive oxygen species) generation.

## 2 MATERIALS AND METHOD

### 2.1 Chemicals and Bacterial Cultures

Dimethyl sulfoxide (DMSO) and nitric acid were purchased from Sigma‐Aldrich. 2, 7‐dichloroflurorescin diacetate (DCFH‐DA) was purchased from Himedia. *Bacillus subtilis* (MTCC No. 441) and *Pseudomonas fluorescens* (MTCC No. 1749) bacterial cultures were procured from the Institute of Microbial Technology (IMTECH), Chandigarh, India. Nutrient agar and nutrient broth were purchased from Himedia. All the aqueous solutions were prepared in Milli‐Q ultra‐pure water (*R* = 18.2 MΩ).

### 2.2 Synthesis and Characterisation of Colloidal Copper Nanoparticles (CuNPs)

Three sets of colloidal copper nanoparticles with per batch yield 0.2 gm (sample A), 0.3 gm (sample B) and 0.4 gm (sample C) were synthesised by the chemical reduction method [26]. As‐synthesised CuNPs were characterised by UV‐visible absorption spectroscopy and photon correction spectroscopy which was reported earlier [26]. Colloidal stability of as‐synthesised nanoparticles was determined by zeta potential measurement. The zeta potential of colloidal CuNPs was measured by phase angle light scattering (PALS) on Brookhaven 90 plus zeta potential analyser. Measurements were carried out at 25°C with gold coated electrodes. For zeta potential measurements, each CuNPs sample was adequately diluted in Milli‐Q ultra‐pure water (*R* = 18.2 MΩ) and well sonicated prior to the measurement. 1.5 ml of CuNPs sample was filled in disposable polystyrene cuvettes. Electrophoretic mobility of the nanoparticles was determined by measuring the Doppler shift in scattered light under constant potential. From this the zeta potential was calculated by using Smoluchowsky’s equation. Transmission electron microscopy (TEM) of as‐synthesised CuNPs was recorded on Philips CM200 transmission electron microscope operated at an accelerating voltage of 200 KV. Samples for TEM measurements were prepared by placing a drop of adequately diluted colloidal nanoparticles on an amorphous carbon‐coated copper grid having a mesh size of 200. Samples were dried overnight under vacuum before the microscopy. Elemental analysis of as‐synthesised colloidal CuNPs was performed by inductively coupled plasma atomic emission spectroscopy (ICP‐AES). Measurements were performed on Leeman labs Prodigy ICP‐AES spectrometer. Before each measurement, the spectrometer was calibrated with the VHG Muti‐element standard. CuNPs samples for ICP analysis were prepared by digesting them with nitric acid [3].

### 2.3 MIC and MBC of CuNPs

Minimum inhibitory concentration (MIC) and minimum bactericidal concentration (MBC) of as‐synthesised CuNPs (sample A, B and C) were determined from standard micro‐dilution method by following the protocols recommended by the National committee of clinical laboratory standards, 2005 (NCCLS) with few modifications [27]. Strains under tests were grown to 0.5 McFarland standard turbidity (10^8^ cfu/ml) [28]. For the determination of MIC, it was first diluted to 10^4^ cfu/ml in nutrient broth. Six sets of test tubes with 10 ml nutrient broth media containing CuNPs in requisite concentration (sample A ‐ 20 to 50 µg/ml, sample B ‐ 20 to 100 µg/ml and sample C ‐ 40 to 70 µg/ml) were prepared (Table S1). Each tube was inoculated with 10^4^ cfu/ml of the respective strain and incubated for 24 h at 37°C for *Bacillus subtilis* and at 30°C for *Pseudomonas fluorescens*. MIC of CuNPs was determined for each strain by measuring their optical density (OD) at 600 nm. The bacterial cultures without the treatment of CuNPs was used as a control. Each experiment was performed in triplicates. To determine MBC, 50 µL aliquots from each test tube used for the determination of MIC was spread on the nutrient agar plates and incubated for 24 h at 37°C for *B. subtilis* and at 30°C for *P. fluorescens* to check the growth or no growth of organisms [28].

### 2.4 Cellular Leakage

#### 2.4.1 Sugar Leakage

Determination of cytoplasmic leakage is one of the methods used to study the effect of nanoparticles on metabolic activity of bacteria. Bursting of bacterial cells releases their intracellular materials like sugars and proteins. The DNS (dinitrosalicylic acid) method was used to determine the leakage of reducing sugars from bacterial cells (10^7^ cfu/ml) after treating them with requisite concentrations of CuNPs (10–70 µg/ml for *B. subtilis* and 20–60 µg/ml for *P. fluorescens*) [29, 30] (Table S1). The tubes were incubated for 12 h at 37°C for *B. subtilis* and at 30°C for *P. fluorescens*. Tubes incubated without CuNPs were treated as controls. After incubation, they were centrifuged at 12,000 rpm at 4°C for 30 min and immediately frozen at −20°C for further analysis. 1 ml supernatant from each tube was taken and 3 ml of DNS (3,5‐dintrosalicycline acid) reagent was added into it. Tubes were then heated at 90°C for 15 min. After cooling, OD was measured at 540 nm to estimate the leakage of reducing sugar from test samples. A standard curve of glucose was used for estimation of the reducing sugar leakage [29, 30]. Experiments were performed in triplicates.

#### 2.4.2 Protein Leakage

Folin‐lowry method was used to determine the protein leakage from the bacterial strains treated with CuNPs [31, 32] The cell density of 10^7^ cfu/ml were treated with 10‐70 µg/ml of CuNPs for *B. subtilis* and 20‐60 µg/ml of CuNPs for *P. fluorescens* and incubated for 12 h at 37°C for *B. subtilis* and at 30°C for *P. fluorescens*. It was then centrifuged at 12,000 rpm for 30 min at 4°C and immediately frozen at −20°C. From each test tube, 1 ml of the supernatant was used for the estimation of protein leakage by measuring their OD at 660 nm. Experiments were performed in triplicates. The standard curve of bovine serum albumin (BSA) was used to estimate the protein leakage. Bacterial cultures without treatment with CuNPs were used as controls.

### 2.5 Estimation of Reactive Oxygen Species (ROS)

Interaction of CuNPs with microorganisms was monitored in terms of ROS generation. It can produce oxidative stress that can cause external or internal cell damage [33]. DCFH‐DA (dichloro‐dihydro‐fluorescein diacetate) has no fluorescence until it enters into the cell through the plasma membrane. Inside the cell, the intracellular esterase converts DCFH‐DA to DCFH. It gets oxidised into a highly fluorescent derivative DCF (2′, 7′‐dichlorofluorescein) when reacted with intracellular ROS. Thus, the fluorescence intensity of DCF is proportional to the amount of intracellular ROS generated. To measure the intracellular ROS levels, 10^7^ cfu/ml of bacterial cultures were treated with requisite quantities of CuNPs (10–70 µg/ml for *B. subtilis* and 20–60 µg/ml for *P. fluorescens*) (Table S1). Cultures were incubated for 12 h at 37°C for *B. subtilis* and at 30°C for *P. fluorescens*. After the incubation, cells were centrifuged at 300× g for 30 min at 4°C. After the centrifugation, the supernatant was treated with 100 μM DCFDA (from 10 mM stock in DMSO) and incubated for 1 h in dark [34]. After the incubation, fluorescence (*λ*ex = 485 nm and *λ*em = 520 nm) was measured on Carry Eclipse fluorescence spectrophotometer. This measured fluorescence intensity is directly proportional to the intracellular ROS.

## 3 RESULTS AND DISCUSSIONS

### 3.1 Characterisation of CuNPs

**FIGURE 1:**
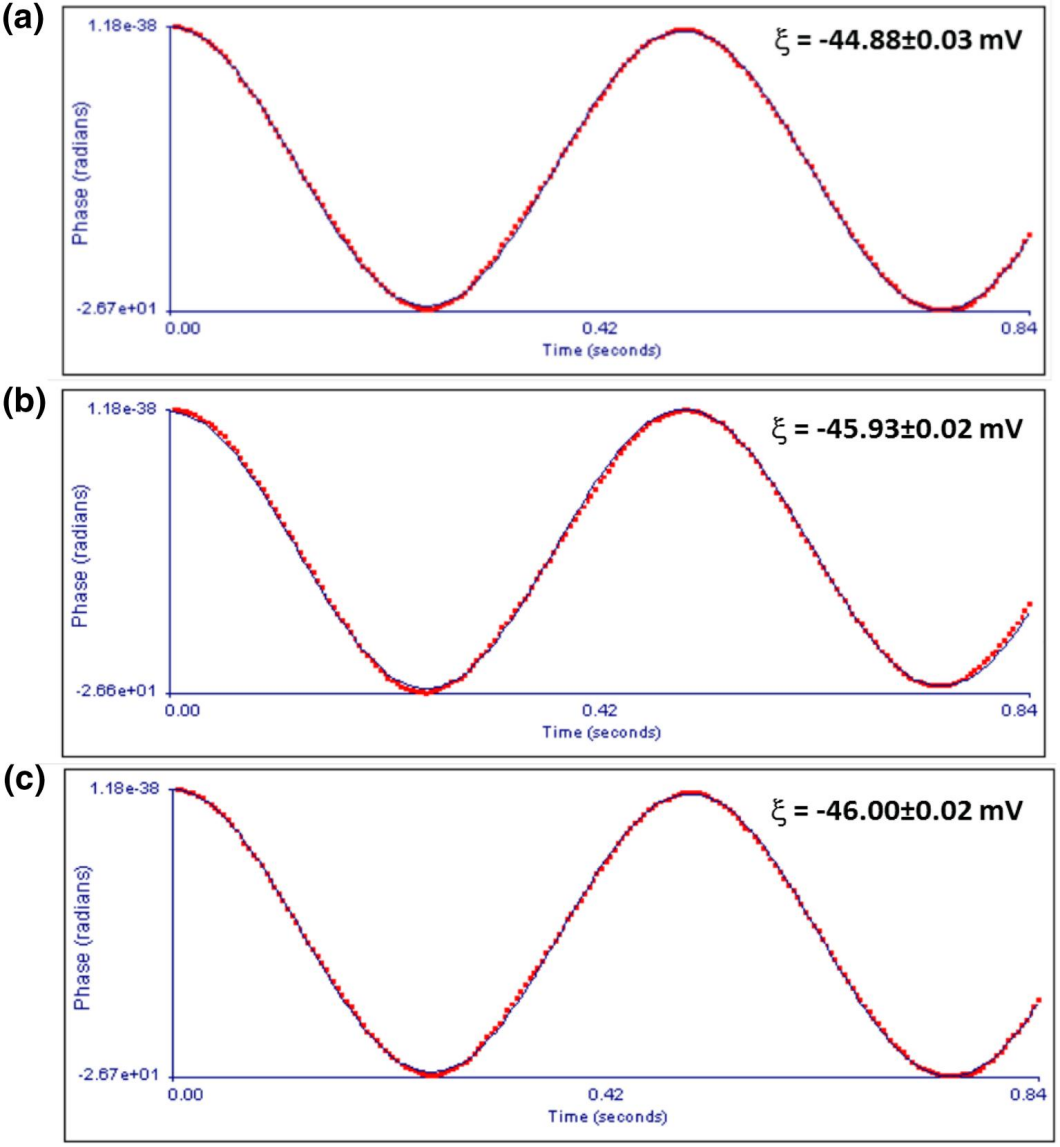
Time dependence of phase angle of CuNPs [Sample A (yield 0.2 gm), B (yield 0.3 gm) and C (yield 0.4 gm)]. From the fits, electrophoretic mobility was determined from which the zeta potential (ξ) of CuNPs was calculated

Three sets of CuNPs (Sample A, B and C) with per batch yield 0.2, 0.3 and 0.4 gm were synthesised by chemical reduction method and characterised by UV‐visible absorption spectroscopy and photon correction spectroscopy (PCS) [26]. As reported previously, irrespective of per batch yield of CuNPs, a single plasmon resonance band at 568 nm with average D_H_ in range of 11‐14 nm was observed [26]. UV visible spectroscopy and PCS study further confirm that the synthesised nanoparticles were in Cu° ionic state and possess monomodal size distribution with symmetric (spherical) morphology [35]. The magnitude of the zeta potential determines the extent of the electrostatic repulsion between the same species constituting the colloid, zeta potential is considered to be an important physical measure for the colloidal stability of nanoparticles [36]. Zeta potential of as‐synthesised CuNPs was determined from electrophoretic mobility measured by the phase angle light scattering (PALS). In this technique, Doppler shift of scattered laser beam from the sample was measured at constant potential applied across the metal electrodes. From the best fit of the phase shift data using Smoluchowksy algorithm, the electrophoretic mobility of the nanoparticles was determined and from this the zeta potential was calculated. The raw data for these measurements are presented in Figure 1. Zeta potential thus determined is reported in Table 1.

**TABLE 1:** Important parameters of as‐synthesised CuNPs [Sample A (yield 0.2 gm), B (yield 0.3 gm) and C (yield 0.4 gm)]

Parameters	A	B	C
Nanoparticle yield (gm)	0.2	0.3	0.4
λ_SPR_ (nm)	568	568	568
A _max_	1.53	2.02	2.58
Hydrodynamic particle size (nm)	11.34	12.19	13.7
Physical size (nm)	9.88 ± 0.39	10.94 ± 0.95	11.49 ± 0.75
Zeta potential (mV)	−44.88 ± 0.03	−45.93 ± 0.02	46.00 ± 0.02
Cu concentration (μg/ml)	1147	1601	2873

The zeta potential of CuNPs is −44.88 ± 0.03 mV (sample A), −45.93 ± 0.02 mV (sample B) and 46.00 ± 0.02 mV (sample C). For all the three CuNPs samples, the zeta potential lies between −44.88 mV and −46.00 mV. Any colloid having its zeta potential ≥ ±30 mV is being considered to be stable [36]. Therefore, it was concluded that the as‐synthesised CuNPs colloids exhibits good colloidal stability.

**FIGURE 2:**
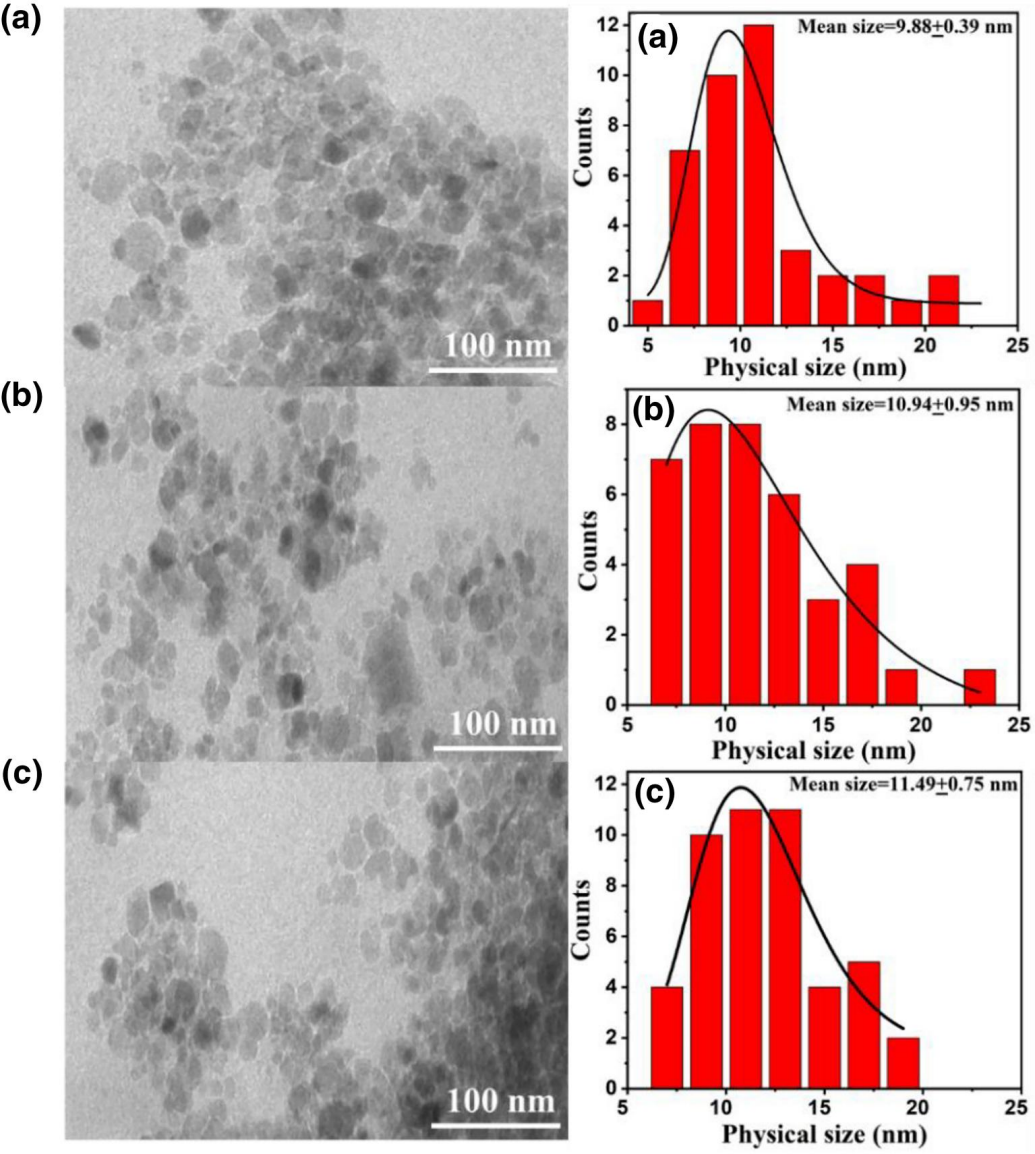
TEM images and their corresponding particle size distribution histograms of CuNPs [Sample A (0.2 gm), B (0.3 gm) and C (0.4 gm)]

TEM images of as‐synthesised CuNPs along with their size distribution histograms were shown in Figure 2. Aggregated nanoparticles with near spherical morphology can be seen in the micrographs of all the three CuNPs. Size distribution histograms were fitted with lognormal particle size distribution function [Equation 1]. From the best fits, the mean physical size of CuNPs was determined (Table 1). The mean physical sizes of CuNPs range between 9.88 and 11.49 nm, which were smaller than their corresponding hydrodynamic particle sizes (D_H_). This marked difference in two sizes is because of hydrodynamic size determined from DLS method includes the CuNPs core and the polymeric PVP shell, while the physical size determined from the TEM microscopy only refers to the CuNPs core [3].

Cu ions concentrations (Table 1) in as‐synthesised colloidal CuNPs were measured by ICP‐AES. Apart from Cu, no other elements were detected by ICP in the tested samples. Cu concentration in CuNPs increases with the increase in their per batch yield. Position of the plasmon resonance band (*λ*_SPR_) and its absorption maximum (*A*_max_) are also determined along with potential (mV) and mean hydrodynamic particle size (D_H_) for all tested CuNPs samples (Table 1). The identical plasmonic characteristic and hydrodynamic particle sizes of synthesised CuNPs further indicates that the scale‐up of nanoparticles' yield from 0.2 gm (sample A) to 0.4 gm (sample C) does not cause any significant deviations in their physical and plasmonic properties.

### 3.2 Ecotoxicity of CuNPs

The ecotoxicity of CuNPs (sample A (0.2 gm), sample B (0.3 gm) and sample C (0.4 gm)) was studied on Gram positive *B. subtilis* (MTCC No. 441) and Gram‐negative *P. fluorescens* (MTCC No. 1749) by determining their minimum inhibitory concentration (MIC) and minimum bactericidal concentration (MBC) [27]. MIC is the lowest concentration of any antimicrobial agent that visually inhibits 99% bacterial growth; whereas MBC is the concentration of antibacterial agent corresponding to which 100% inhibition in bacterial growth is observed. Photographic images of MIC and MBC tests on both the microorganisms are presented in appendix (Fig. S1 and Fig. S2, respectively). For CuNPs sample A (yield 0.2 gm), the MIC and MBC values for both the strains (*B. subtilis* and *P. fluorescens*) are identical, which are 35 ± 5 µg/ml and 40 ± 5 µg/ml, respectively (Table 2).

**TABLE 2:** MIC and MBC of the as‐synthesised CuNPs [Sample A (yield 0.2 gm), B (yield 0.3 gm) and C (yield 0.4 gm)] against *B. subtilis* and *P. fluorescens*

CuNPs	Strains			
	*B. subtilis*		*P. fluorescens*	
	MIC		MIC	
	(μg/ml)	MBC	(μg/ml)	MBC
A (0.2 gm)	35 ± 5	40 ± 5	35 ± 5	40 ± 5
B (0.3 gm)	40 ± 5	60 ± 5	40 ± 5	45 ± 5
C (0.4 gm)	60 ± 5	70 ± 5	55 ± 5	60 ± 5

This indicates that Sample A of CuNPs imparts identical ecotoxicity on both the tested microorganisms irrespective whether the tested strain is Gram positive or Gram negative. In contrast to this, CuNPs of sample B show greater ecotoxicity on Gram positive *B. subtilis* as compared to Gram‐negative *P. fluorescens*. MBC (Table 2) value for *B. subtilis* is higher than that of *P. fluorescens*. In case of CuNPs of sample C, both MIC and MBC values are higher for *B. subtilis* as compared to *P. fluorescens*.

With the increase in the hydrodynamic size of CuNPs (from Sample A to Sample C), the ecotoxicity of nanoparticles decreases in case of Gram‐negative strain of *P. fluorescens*. No such systematic size dependence is observed in Gram positive *B. subtilis*. The difference between the Gram‐positive and Gram‐negative strains is in the cell membrane structure and its composition. The Gram positive bacteria have thick peptidoglycan layer (20–30 nm) and linear polysaccharides chains cross linked by short peptides that make their cell walls rigid and difficult to penetrate by the nanoparticles; where as in Gram‐negative bacteria cell walls are made up of thin layer of peptidoglycan (8–12 nm) and a layer of lipopolysaccharides. They lack the strength and rigidity which make them prone to nanoparticles [37]. Because of this difference in the cell wall thickness in two strains penetration of nanoparticles and subsequent interaction is more pronounced in Gram‐negative strains (*P. fluorescens*) and hence they are more sensitive to nanoparticles as compared to Gram positive *B. subtilis* [37]. Similar results were reported by Yerukala and Bokka where *P. fluorescens* was more sensitive towards copper nanoparticles than *B. subtilis* due to the difference in their cell membrane structure [24].

Another possible mechanism involves elution of Cu^2+^ ions from nanoparticles, which gets absorbed on bacterial cell membrane and damages the membrane either by altering their enzyme functions or by solidifying proteins [38]. To further prove this, the growth curve analysis was performed for both strains. Growth profiles of both the strains with and without CuNPs were recorded for 10 h, which are presented in the appendix (Figure S3). Irrespective of the strain, the growth profiles in the presence of CuNPs show reduction in the growth rates with the increasing CuNPs concentration. This is more pronounced in Gram‐negative strain (*P. fluorescens*) than in Gram positive strain (*B. subtilis*).

### 3.3 Cytoplasmic Leakage

**FIGURE 3:**
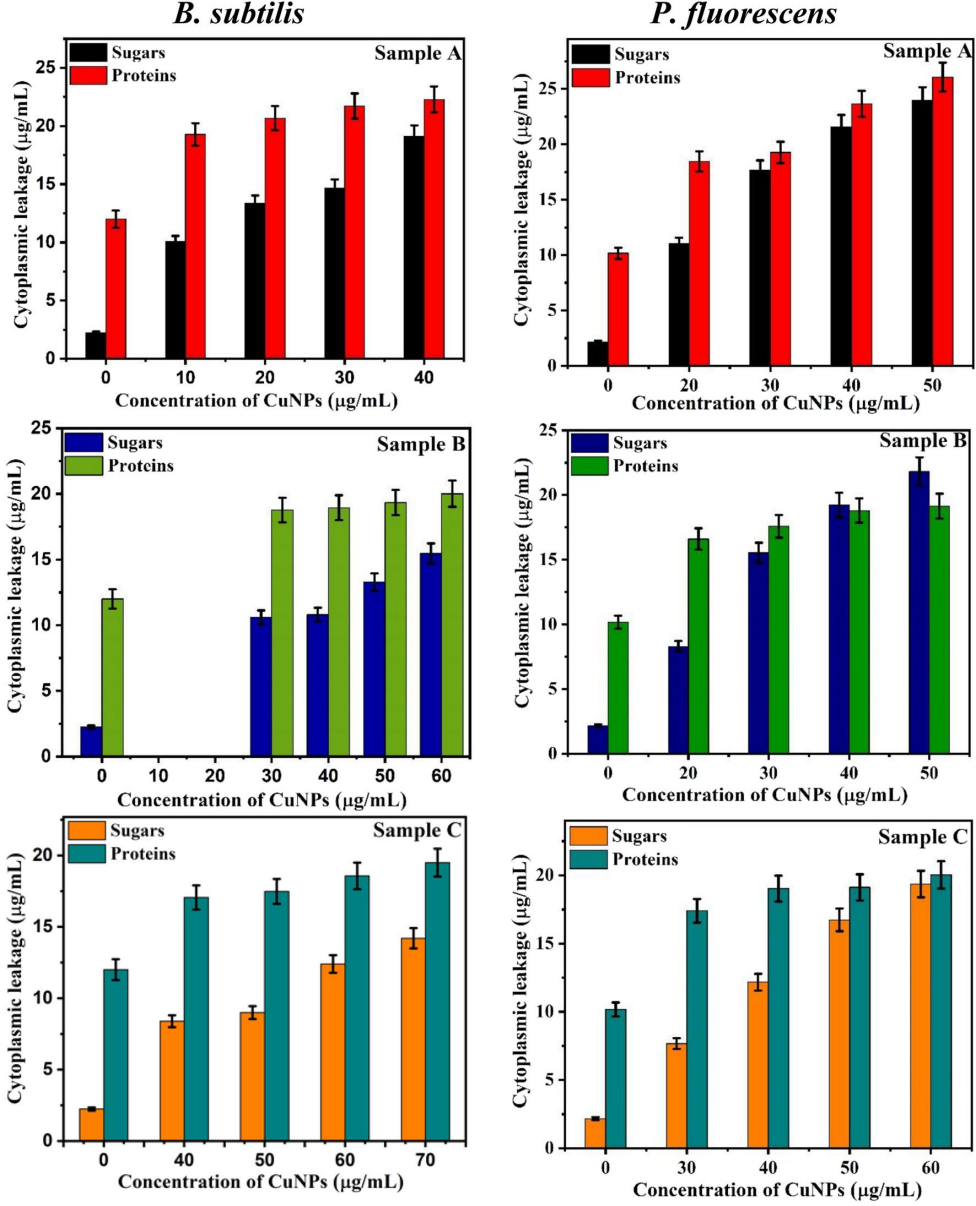
Effect of CuNPs [Sample A (yield 0.2 gm), B (yield 0.3 gm) and C (yield 0.4 gm)] on cellular leakage of sugars and proteins after 12 h incubation. CuNPs at 0 μg/ml represents negative control (i.e. media + bacteria). Values are average of three replicates and *error bars* represents standard deviation

To check the effect of CuNPs on metabolic activities of bacteria, cytoplasmic leakage of intracellular macromolecules (sugars and proteins) was studied. Amount of sugar and protein leakage from *P. fluorescens* and *B. subtilis* in the presence of CuNPs (Sample A, B and C) is shown in Figure 3. The sugar and protein leakage is minimum in cultures not treated with CuNPs (controls). With the increase in the CuNPs concentrations, the amount of sugar and protein leakage from both strains increases. At highest tested CuNPs dose (i.e. MBC), the leakage was more pronounced in Gram‐negative strain *P. fluorescens* as compared to the Gram positive strain *B. subtilis*. Amongst the three tested CuNPs, the leakage is highest for Sample A followed by sample B and sample C. This is in correlation with their hydrodynamic sizes, which is lowest for sample A followed by sample B and sample C. Particles with smaller size be able to penetrate more through the bacterial cells and disrupts the biochemical activities. Thick cell wall in Gram positive strains *B. subtilis* functions as a barrier against antimicrobial agents. These observations are in a good correlation with the results of MIC and MBC tests.

Yuan et al. [34] and Saratale et al. [39] have also observed higher sugar and protein leakage in Gram‐negative strains as compare to Gram positive strains when treated with silver nanoparticles in a concentration‐dependent manner indicated possible antibacterial mechanism by cytoplasmic leakage. Several reports are found in literature where increased sugar leakage from clinical pathogens has been observed [39, 40, 41]. Earlier reports also suggest that metallic nanoparticles disrupts the permeability of bacterial cell membranes, affects membrane transport system and induces release of cellular macromolecules [39, 40, 41].

### 3.4 Reactive Oxygen Species (ROS)

**FIGURE 4:**
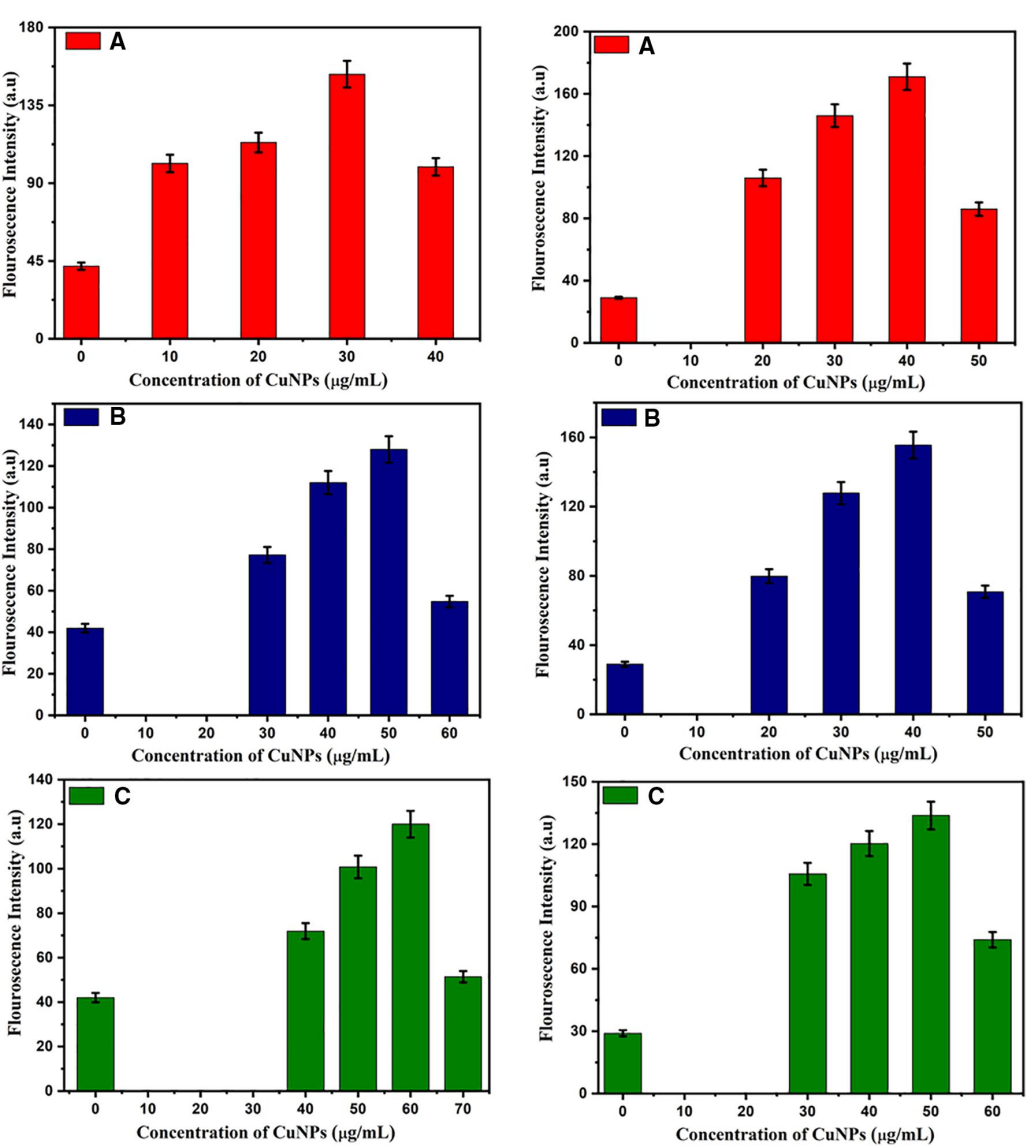
Fluorescence intensity representing amount of intracellular ROS generation in bacterial stravvins after their treatment with CuNPs [Sample A (yield 0.2 gm), B (yield 0.3 gm) and C (yield 0.4 gm)]. CuNPs at 0 μg/ml represents negative control (i.e. media + bacteria). Values are average of three replicates and *error bars* represents standard deviation

Oxidative stress produced by reactive oxygen species (ROS) in *B. subtilis* and *P. fluorescens* after their treatment with CuNPs was measured in terms of fluorescence produced by DCF (Figure 4). Strains treated with CuNPs show higher ROS levels as compared to untreated cells. Irrespective of strains and CuNPs samples, the ROS levels increases with the increase in CuNPs concentration till its MIC.

Beyond this concentration (i.e. at MBC), the ROS level quenched in both the organisms for all the three CuNPs samples. This might be because of bacterial cell deaths caused by higher oxidative stress at MBC. The ROS levels measured in terms of fluorescence intensity of DCF generated in Gram‐negative *P. fluorescens* after their treatment with CuNPs (at MIC) is higher than that measured in Gram positive *B. subtilis*. This difference in ROS levels can be ascribed to differential internalisation of CuNPs in both the strains because of the difference in their cell membrane structures.

Yuan et al. [34] and Kim et al. [40] have also reported similar observations; where silver nanoparticles induce greater ROS levels in Gram‐negative strains as compared to Gram positive strains. Further, we have observed decrease in ROS levels in both the strains with the increase in the hydrodynamic size of CuNPs (from sample A to sample C). This might be because of the greater penetration power of smaller size nanoparticles. This observation is in line with those observed in MIC, MBC and cytoplasmic leakage cell damage when subject to stress like cold, heat and toxins [42, 43].

Nanoparticle‐microorganism interaction can produce four different types of ROS such as hydrogen peroxide (H_2_O_2_), hydroxyl radicals (OH), hydroperoxyl radicals and super oxide ions (O_2_). In literature, it has been reported that CuNPs can produce all four types of reactive oxygen species and hence can cause greater intracellular damage [44]. CuNPs induce oxidative stress (ROS) can induce cell apoptosis by DNA, nucleic acid and proteins damage, or thorough loss of cell membrane integrity and through intracellular respiratory failure [40, 45].

## 4 CONCLUSION

As‐synthesised CuNPs showed high ecotoxicity on tested soil bacteria even at low concentrations through loss of cell viability by leakage of intracellular proteins and sugars and increased oxidative stress by generation of ROS. CuNPs induced greater toxicity on Gram‐negative (*P. fluorescens*) strains as compared to Gram positive (*B. subtilis)* bacteria mainly due to differences in cell wall structure and its composition as evidenced by MIC, MBC, cytoplasmic leakage and ROS assays. CuNPs (sample A) with smallest hydrodynamic particle size of 11.34 nm exhibited highest antibacterial activity owing to greater membrane permeability and higher surface area. Therefore, unintentional release of CuNPs in soil is a matter of great concern because of their negative impact on soil microbial communities. In situ studies are necessary to see the overall impact of CuNPs on soil health and its micro‐ecosystem, since, this has direct impact on nutrient cycling by various microorganisms.

## Conflicts of Interest

There are no conflicts to declare.

## Supporting information

Supplementary Material
