# Supplementary material for: Ecotoxicity of as‐synthesised copper nanoparticles on soil bacteria
Source: IET Nanobiotechnol. 2021 Mar 30;15(2):236–45. doi: 10.1049/nbt2.12039 (PMC8675774; doi:10.1049/nbt2.12039)
Supplement: Supplementary file 1 — Supplementary Material [file NBT2-15-236-s001.docx]

**Appendix**

**Table S1:** Test concentrations and corresponding volume of colloids (Sample A, B and C) used in MIC, cellular leakage and ROS assay.

| **Sample A** | | **Sample B** | | **Sample C** | |
| --- | --- | --- | --- | --- | --- |
| Test concentrations  (μg/mL) | Volume used^*^ (μL) | Test concentrations  (μg/mL) | Volume used^*^ (μL) | Test concentrations  (μg/mL) | Volume used^*^ (μL) |
| 10 | 0.09 | 20 | 0.12 | 30 | 0.10 |
| 20 | 0.17 | 25 | 0.15 | 40 | 0.14 |
| 25 | 0.22 | 30 | 0.18 | 45 | 0.15 |
| 30 | 0.26 | 35 | 0.22 | 50 | 0.17 |
| 35 | 0.31 | 40 | 0.25 | 55 | 0.19 |
| 40 | 0.35 | 45 | 0.28 | 60 | 0.21 |
| 45 | 0.39 | 50 | 0.31 | 65 | 0.22 |
| 50 | 0.43 | 60 | 0.37 | 70 | 0.24 |
|  |  | 80 | 0.49 |  |  |
|  |  | 100 | 0.62 |  |  |

*Calculations were based on the total volume = 10 mL for MIC, Cytoplasmic leakage and ROS analysis. Stock concentrations were Sample A = 1147 μg/mL, Sample B = 1601 µg/mL and Sample C = 2873 µg/mL.

**Table S2**: Concentrations of CuNPs for which growth kinetics have been determined

| **Bacteria** | **CuNPs concentrations (µg/mL)** | | |
| --- | --- | --- | --- |
|  | **Sample A** | **Sample B** | **Sample C** |
| *B. subtilis* | 10, 20, 30 & 40 | 10, 30, 50 & 70 | 20, 40, 60 & 80 |
| *P. fluorescens* | 15, 25, 35 & 45 | 20, 30, 40 & 50 | 25, 40, 55 & 65 |

**MIC Test:**


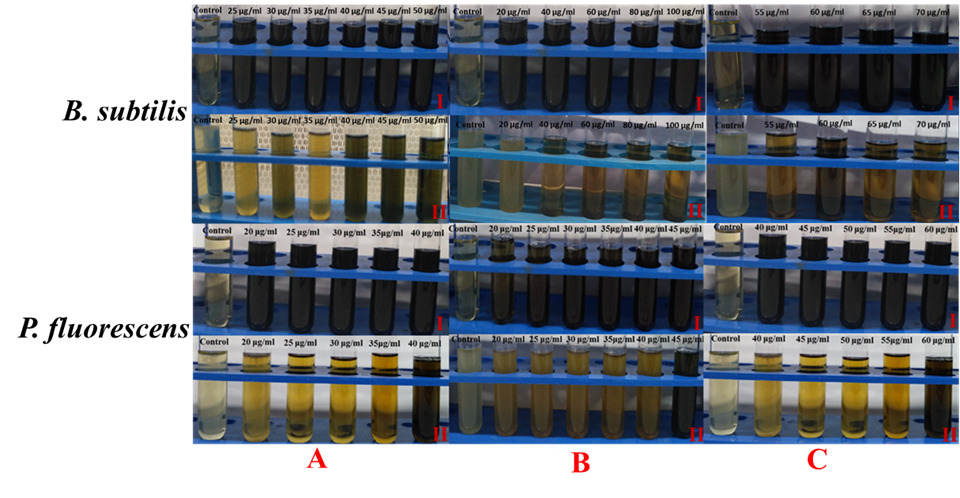


**Fig. S1.** Photographic view of MIC (I - before incubation and II - after incubation) of samples A, B and C*.* Order of the tubes from left to right in *B. subtilis*; (1). Control (i.e., medium + bacterial strain); (2). 25-50 µg/mL CuNPs; (3). 20-100 µg/mL CuNPs; (4) 50-70 µg/mL CuNPs. Order of the tubes from left to right in *P. fluorescens*; (1). Control (i.e., medium + bacterial strain); (2). 20-40 µg/mL CuNPs; (3). 20-45 µg/mL CuNPs; (4) 40-60 µg/mL CuNPs.

**MBC Test:**

**
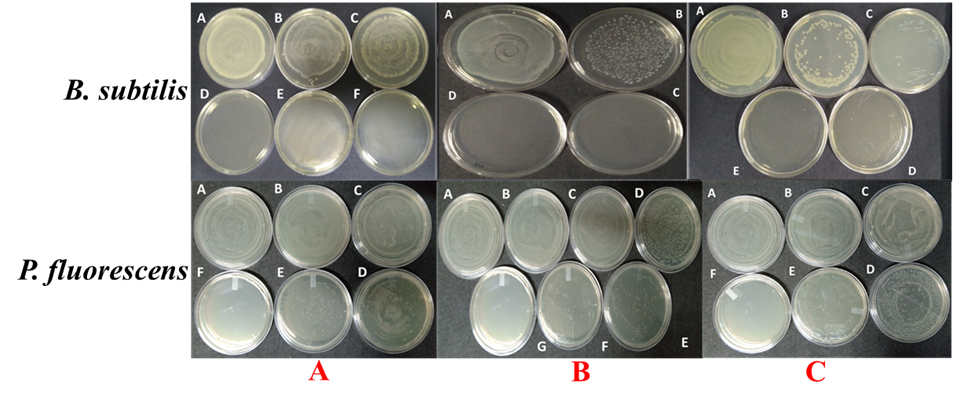
**

**Fig. S2.** Photographic view of MBC test*.* Order of the plates from left to right in *B. subtilis* in sample A; (A). Control (i.e., agar + bacterial strain); (B). 25 µg/mL CuNPs; (C). 35 µg/mL CuNPs; (D) 40 µg/mL CuNPs; (E) 45 µg/mL CuNPs; (F) 50 µg/mL CuNPs. Order of the plates from left to right in *B. subtilis* in sample B; (A). Control (i.e., agar + bacterial strain); (B). 20 µg/mL CuNPs; (C). 40 µg/mL CuNPs; (D) 60 µg/mL CuNPs. Order of the plates from left to right in *B. subtilis* in sample C; (A). Control (i.e., agar + bacterial strain); (B). 50 µg/mL CuNPs; (C). 55 µg/mL CuNPs; (D) 60 µg/mL CuNPs; (E) 70 µg/mL CuNPs. Order of the plates from left to right in *P. fluorescens* in sample A; (A). Control (i.e., agar + bacterial strain); (B). 20 µg/mL CuNPs; (C). 25 µg/mL CuNPs; (D) 30 µg/mL CuNPs; (E) 35 µg/mL CuNPs; (F) 40 µg/mL CuNPs. Order of the plates from left to right in *P. fluorescens* in sample B; (A). Control (i.e., agar + bacterial strain); (B). 20 µg/mL CuNPs; (C). 25 µg/mL CuNPs; (D) 30 µg/mL CuNPs; (E) 35 µg/mL CuNPs; (F) 40 µg/mL CuNPs; (G) 45 µg/mL CuNPs. Order of the plates from left to right in *P. fluorescens* in sample C; (A). Control (i.e., agar + bacterial strain); (B). 40 Sµg/mL CuNPs; (C). 45 µg/mL CuNPs; (D) 50 µg/mL CuNPs; (E) 55 µg/mL CuNPs; and (F) 60 µg/mL CuNPs.

**Growth kinetics:**

| ***P.*** ***fluorescens***  ***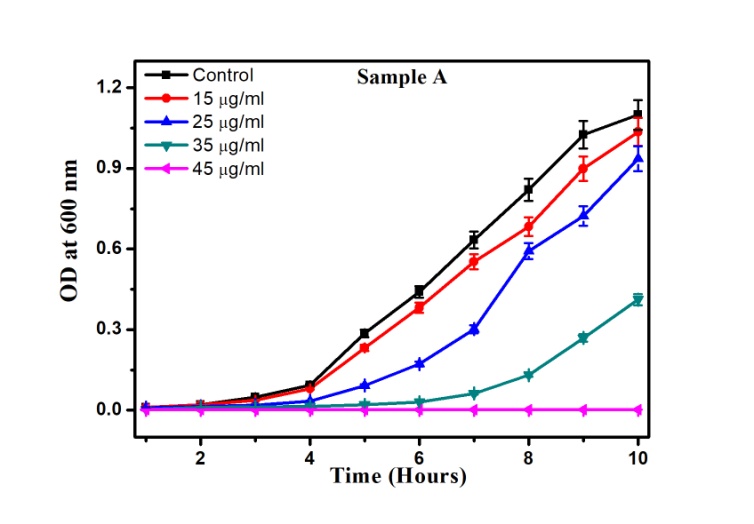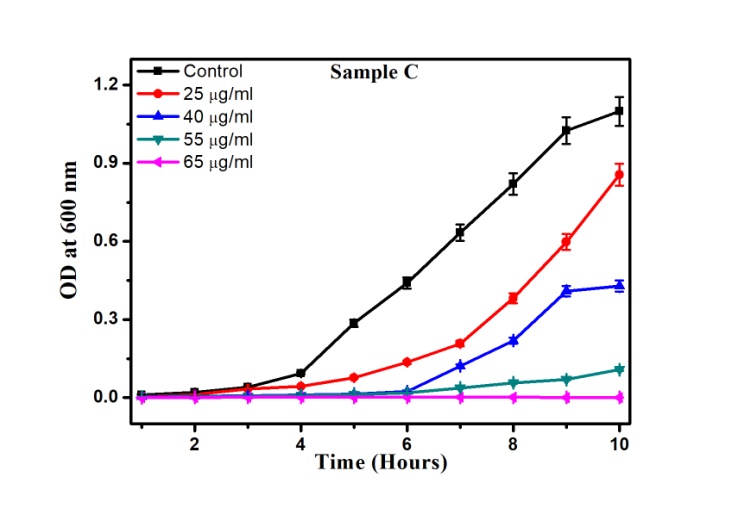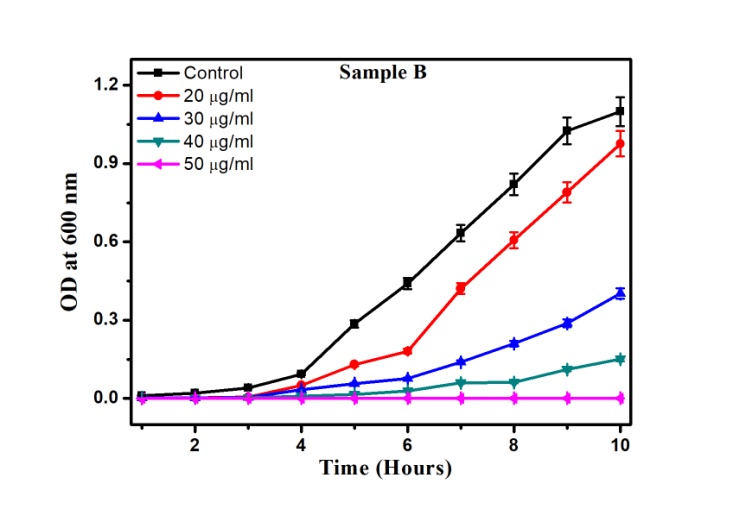*** | ***B. subtilis***  ***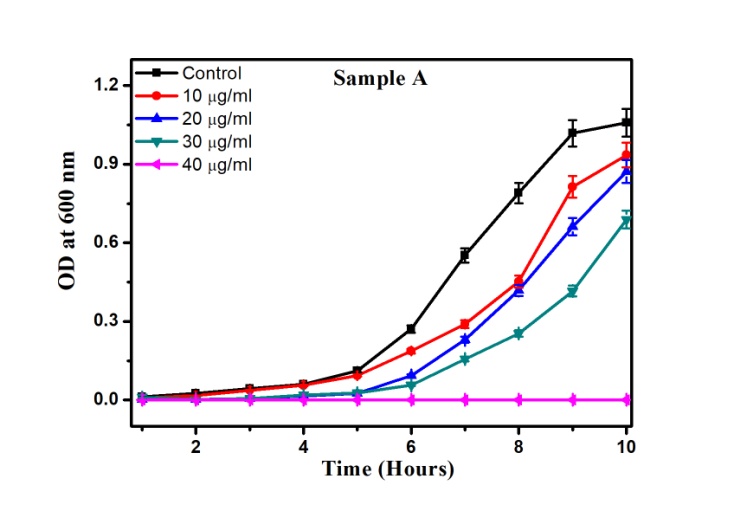***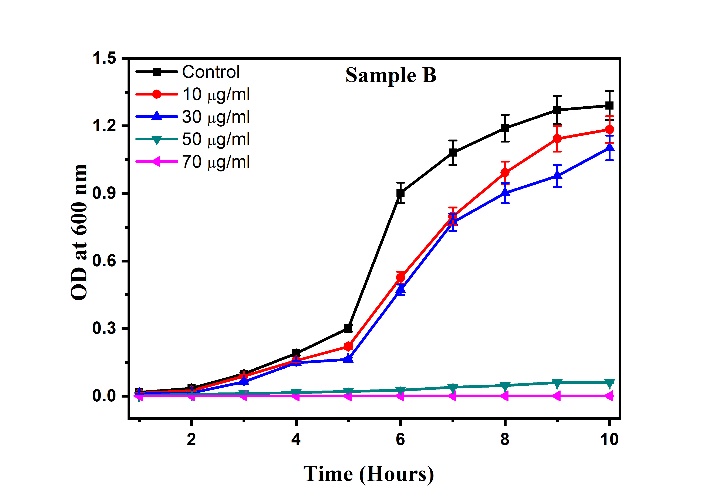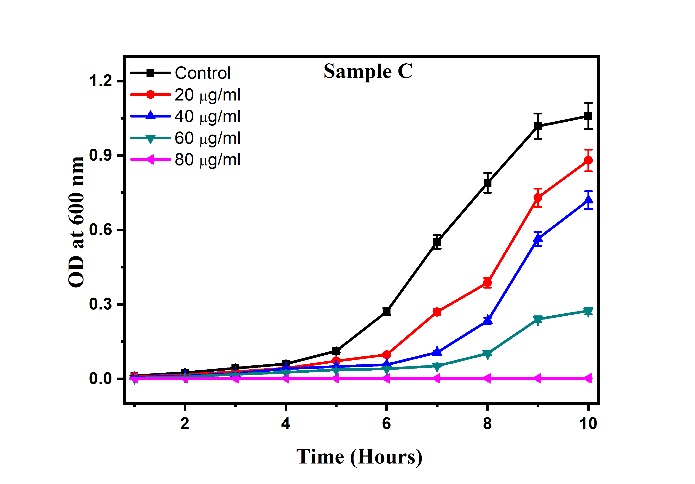 |
| --- | --- |

**Figure S3:** Growth curves of *B. subtilis* and *P. fluorescens* after treating them with CuNPs. Each point on the curve is average of three replicates and *error bars* represents standard deviation.

Evaluation of bacterial growth kinetics of *B. subtilis* and *P. fluorescens* in the presence of CuNPs (three samples namely A, B and C with per batch yield 0.2, 0.3 and 0.4 gm) was performed by following the protocols developed by Ruparelia *et al*. Freshly grown culture (OD = 0.01; 10^8^cfu/mL) was used to examine the growth pattern of bacteria in broth medium. 1 % inoculum was added into 50 mL nutrient broth with requisite concentrations of CuNPs in each flasks. Growth kinetics was studied for four concentrations (two below MIC, one at MIC and one above MIC) for each CuNPs samples. For each bacterium, these concentrations are listed in Table 1. Following this, each flask was incubated at 37 ^o^C for *B. subtilis* and at 30 ^o^C for *P. fluorescens*. The growth kinetics was determined by recording the OD at 600 nm for 0 - 10 h at an interval of 1 h. For reference growth kinetics of positive (culture and media) and negative (media and CuNP) controls have also been determined. The measured OD values have been corrected with corresponding negative controls.

The control showed normal growth pattern till 10 hours but there was alterations of growth pattern in treated ones with the extension of their lag phases in each sample as shown in Figure S3. The growth curve data showed that with increasing concentration of CuNPs the bacteria growth starts decline At concentration below MIC, the growth rate is not highly inhibited as compared to control but at MIC concentration the growth rate is highly inhibited nearly 80 % and almost declined after 7 or 8 h in all three samples as compared to control. At much higher concentration, MBC the rate of bacterial growth is almost zero. There was no growth at higher concentration in both bacteria with each CuNPs samples. These results showed that toxic effects of each CuNPs sample irrespective of its yield was enough to outpace the speed of reproduction of bacteria and eventually lead to cell death. Again the growth curves of gram negative showed faster growth inhibition than gram positive bacteria.
